# Mechanically and Thermally Induced Degradation and Modification of Cereal Biopolymers during Grinding

**DOI:** 10.3390/polym11030448

**Published:** 2019-03-08

**Authors:** Sabina Paulik, Mario Jekle, Thomas Becker

**Affiliations:** Technical University of Munich, Institute of Brewing and Beverage Technology, Research Group Cereal Technology and Process Engineering, 85354 Freising, Germany; sabina.jakobi@tum.de (S.P.); tb@tum.de (T.B.)

**Keywords:** milling, physical flour modification, grinding, wheat starch, starch damage, hydration properties

## Abstract

It is presumed that structural and functional alterations of biopolymers, which occur during grinding, are caused by a mechanical modification of polymers. As a result, thermally induced changes of flours are neglected. In this study, the impact of thermo-mechanical stress (TMS), as occurring during general grinding procedures, was further differentiated into thermal stress (TS) and mechanical stress (MS). For TS, native wheat flour, as well as the purified polymers of wheat—starch and gluten—were heated without water addition up to 110 °C. Isolated MS was applied in a temperature-controlled ultra-centrifugal grinder (UCG), whereby thermal and mechanical treatment (TMS) was simultaneously performed in a non-cooled UCG. TS starch (110 °C) and reference starch did not show differences in starch modification degree (2.53 ± 0.24 g/100 g and 2.73 ± 0.15 g/100 g, AACC 76-31), gelatinization onset (52.44 ± 0.14 °C and 52.73 ± 0.27 °C, differential scanning calorimetry (DSC)) and hydration properties (68.9 ± 0.8% dm and 75.8 ± 3.0%, AACC 56-11), respectively. However, TS led to an elevated gelatinization onset and a rise of water absorption of flours (Z-kneader) affecting the processing of cereal-based dough. No differences were visible between MS and TMS up to 18,000 rpm regarding hydration properties (65.0 ± 2.0% dm and 66.5 ± 0.3% dm, respectively). Consequently, mechanical forces are the main factor controlling the structural modification and functional properties of flours during grinding.

## 1. Introduction

Wheat flours display unique interactions of water, gluten proteins and starch fractions during dough preparation and bread making, which enable the production of porous foods such as white bread and cakes [1]. Wheat flour quality is often described by the degree of starch damage (hereinafter called the starch modification degree (SMD)) [2,3,4]. However, the term starch damage comprises poorly defined structural as well as functional modifications of flours, such as the water retention capacity [5], that occur during grinding. (Post)-Grinding or extrusion of cereals resulted in enhanced hydration properties of physically modified flours, which can lead to desired higher bread weight [6], but also to an adverse decrease in loaf volume [7,8] by creating altered dough properties [9]. A detailed elucidation of underlying mechanisms has not been completed yet, since different types of physical forces take place simultaneously during the modification process and affect biopolymer modification on the molecular, nanoscopic and microscopic scale.

**Figure 1:**
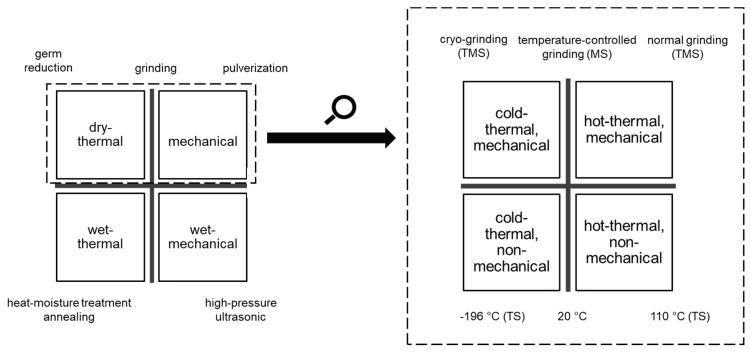
Physical modification of cereals; left: physical stresses in fluid and dry media, right: differentiation of physical stresses in dry media. TMS = thermal-mechanical stress, MS = mechanical stress.

The physical modification of cereal polymers can thereby occur under dry conditions or in the presence of water through the treatment of polymer suspensions (Figure 1, left).

Although a lot of research was related to physical starch modification in suspensions, as heat moisture treatments (HMT), annealing and ultrasonic treatments [10,11,12,13,14,15], only few studies have dealt with modification techniques, as high-pressure treatment, of cereal materials with low moisture content (≤14%) [16]. Thereby, the type of structural and consequently, functional characteristics of flours strongly depend on the modification technique and the parameters used. While HMT is known to increase the gelatinization onset and to decrease the gelatinization enthalpy with elevated moisture content of the sample, the size and surface of starch granules are not altered [11]. However, ultrasonically treated starch became porous and starch granules were disrupted after the treatment [14,15]. This complicates a clear definition of starch modification, since modifications occur on different structural levels of starch, which depends on the applied physical forces.

The most common physical modification process—the grinding procedure of cereals—combines thermal and mechanical forces [17]. Nevertheless, a sharp differentiation between a mechanically and thermally induced flour modification has not been made and flour modifications during grinding were generally referred to a mechanically induced modification of flours. Thereby, previously studies showed that prolonged heat-treated whole grains or flours exhibited changes in structural conformation and functionality [18,19], as protein denaturation [19] and partial gelatinization [20,21]. However, the effects of shorter dry heat treatments on flour characteristics, as applied during grinding, have not been analyzed so far, although the temperature of ground rice reached 75 °C and 88 °C for hammer mill and high-speed impact mill, respectively.

As a result, the distinction between thermally and mechanically induced alterations is a prerequisite to purposively modify the functional properties of the natural biopolymer starch during the grinding procedure. The physical modification during grinding presents a comparably cheap method and offers benefits compared to a chemical modification, since physically modified starch does not have to be declared and is free of chemical agents [22]. 

To enable the elucidation of the impact of isolated thermal and mechanical forces during grinding on polymer modification, flour (wheat, rye, rice) as well as gluten and wheat starch were subjected either to a thermal treatment, to a combined thermal and mechanical treatment (heat and impact/shear) in a grinder, or to a mechanical treatment (impact/shear) by a temperature-controlled grinding procedure. Subsequent analysis of hydration properties (WRC, AACC 56-11), starch modification degree (AACC 76-31), particle size distribution and characteristics of starch crystal melting provided information concerning the modification on structural and functional levels of cereal biopolymers. Dough made from (modified) flours were analyzed by a Z-kneader system to gain detailed knowledge of correlations between flour treatments and functionality. The aim of the study was to differentiate between mechanical and thermal effects on flour during grinding in order to achieve a targeted modification of wheat flours or to standardize the grinding process. Based on this knowledge, physically modified flours or starches can be purposively deployed in the food industry and replace chemical modified starches. 

## 2. Materials and Methods

### 2.1. Physical Treatments of Raw Materials

Pure variety wheat grain ‘Akteur’ (donated by Kunstmühle, Buchloe, Germany; ground by Rosenmühle GmbH, Ergolding, Germany;), wheat starch (Hamstarch A, Jaeckering Muehlen- und Naehrmittelwerke GmbH, Hamm, Germany) and gluten (Kroener-Staerke GmbH, Ibbenbueren, Germany) were used in this study.

Furthermore, selected experiments were performed on a second wheat flour variety (Rosenmühle GmbH, Ergolding, Germany), rice flour (Mueller’s Muehle GmbH, Gelsenkirchen, Germany) and rye flour (Rosenmühle GmbH, Ergolding, Germany). Moisture content (AACC- 44-01) of reference samples was 11.96 ± 0.03%, 13.87 ± 0.06%, 9.50 ± 0.04%, 13.70 ± 0.02%, 12.49 ± 0.02% and 12.26 ± 0.06%, respectively. The substances were exposed to either mechanical stress (MS), thermo-mechanical-stress (TMS) or thermal stress (TS). To achieve a thermal modification, bio-materials were distributed in thin layers (<5 mm) on baking parchment and thermal stress (TS) was applied for 13 min at 20 °C, 40 °C, 65 °C, 80 °C, 95 °C, and 110 °C in a baking oven. Thermo-mechanical modification was achieved by a non-temperature controlled impact grinder (ZM 200, Retsch, Haan, Germany). Flour samples were ground (at room temperature) at rotational speeds of 0, 6000, 9000, 12,000, 15,000 and 18,000 rpm using a 250 µm mesh size sieve, resulting in extensive heat development and a temperature rise. Mechanical treatment (MS) of flours was achieved by using pre-cooled flour (−18 °C). The grinding procedure (0–18,000 rpm, 250 µm mesh size sieve) was performed in a cooled operational room (+ 4°C) for MS, so that flour temperature did not exceed 60 °C during mechanical treatment. After modification, samples were hermetically sealed and stored at 20 °C ± 2 °C in the dark until the following analysis. All modification procedures were performed in triplicates (*n* = 3).

### 2.2. Particle size Distribution

Changes in particle size were evaluated using Mastersizer 3000 (static light scattering, Aero S Unit, Malvern Instruments Ltd., Worcestershire, UK). The dry dispersion unit enabled the measurement of modified flours, gluten and starch excluding a particle hydration. To calculate the particle size, the measurement principle ‘Mie Theory’ with a refractive index of 1.54 and the general-purpose mode was applied.

### 2.3. Starch Modification Degree

The starch modification degree was measured by means of an enzyme assay kit (approved method AACC 76-31, starch damage, Megazyme International Ltd., Ireland). The method determines the percentage of starch granules (on 14% moisture basis) in flour and starch hydrolyzed amylases within a defined time range. Calculation was performed using the following Equation (1):

SMD = ΔE × F × 90 × (1/1000) × (100/W) × (162/180)(1)

with SMD = starch modification degree (%), ΔE = absorbance (reaction) read against the reagent blank, F = (150 (μg of glucose))/(absorbance of 150 μg of glucose), W = sample weight (mg).

### 2.4. Starch Crystal Melting (Gelatinization Properties)

Determination of gelatinization properties of starch and flour samples were carried out using a differential scanning calorimeter (DSC) (Pyris Diamond, PerkinElmer, Waltham, MA, USA) equipped with an Intracooler 2P cooling system. Calibration was performed with indium and *n*-decan for a temperature range from +20 to +95 °C. Each sample was mixed with deionized water (flour: water ratio = 1:3), filled in DSC pans (20–35 mg) and hermetically sealed. After 2 min of equilibration at 25 °C, samples were heated to 95 °C at 10 K/min in a nitrogen atmosphere. The gelatinization onset (*T*o), peak (*T*p), and conclusion temperatures (*T*c), and the enthalpy of gelatinization (Δ*H*) were determined using the Pyris software.

### 2.5. Water Retention Capacity (WRC)

Hydration properties of flour, gluten and starch were analyzed in accordance with the approved method of the AACC 56–11.02 (solvent retention capacity with water = water retention capacity) for reference and modified samples. WRC was calculated as the weight of the water hold by wheat flour samples after 10 min of centrifugation at 1000× *g* based on dry mass.

### 2.6. Dough Properties (Z-Kneading System)

Dough was with (thermally modified) wheat flour (100 parts) and distilled water. According to the AACC method 54.21 a torque measuring Z-kneader system (DoughLab; Perten Instruments, Germany) was used to determine the optimum water addition of flours. The optimum was reached when torque was between 480 and 520 FU (farinograph units).

### 2.7. Statistical Analysis

The statistical analysis was performed with JMP^®^ Pro (Version 12.2.0, JMP Software, © SAS Institute Inc). To estimate the proportion of variation in the response, coefficient of multiple determination (R^2^) was calculated as (sum of squares (model))/(sum of squares (total)).

## 3. Results and Discussion

### 3.1. Particle Size Distribution

**Figure 2:**
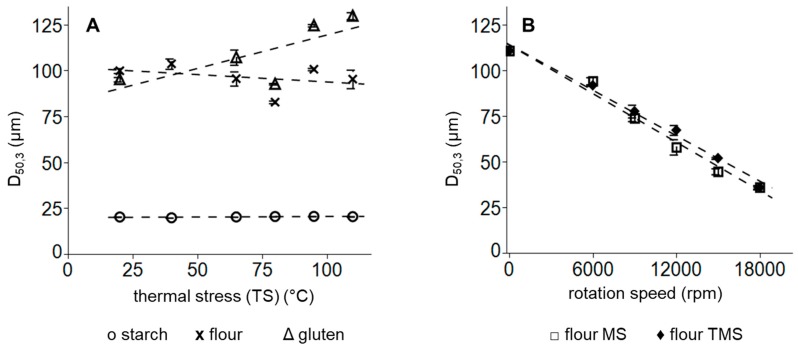
Mean particle size distribution after (**A**) exclusively thermal treatment of flour x, starch O and gluten Δ and (**B**) after mechanical treatment with thermal stress (TMS) ♦ or without thermal stress (MS) ≤ of wheat flours. Error bars represent standard deviations; *n* = 3.

Particle size distribution (PSD) of flours provides important knowledge to predict the functional behavior of modified flours. In general, wheat flours show a three-modal distribution: the first peak (<10 µm) is formed by small, spherical B starch granules, the second peak (around 10–41 µm) by disc-shaped A starch granules and the largest peak (41–300 µm) by starch granule-protein agglomerates [23], whereby 89%–98% of the particle volume distribution were allocated to the fractions ranging from 10–41 µm and 41–300 µm [23,24]. However, B granules represents the greatest fraction in the number distribution of wheat flours [25]. The value D_50,3_ describes the medium, volume particle size of each flours and decreases triggered by an increase in the extent of certain physical treatments, such as high-pressure application [26]. In this study, an isolated thermal treatment of wheat flour or pure starch did not lead to alterations of the mean volume particle size of the samples (compare Figure 2A). However, the formation of gluten aggregates was observed, when temperatures exceeded 90 °C. These findings can be explained by the formation of disulphide bonds, as shown by investigations on the low moisture gluten of Weegels et al. [27].

**Figure 3:**
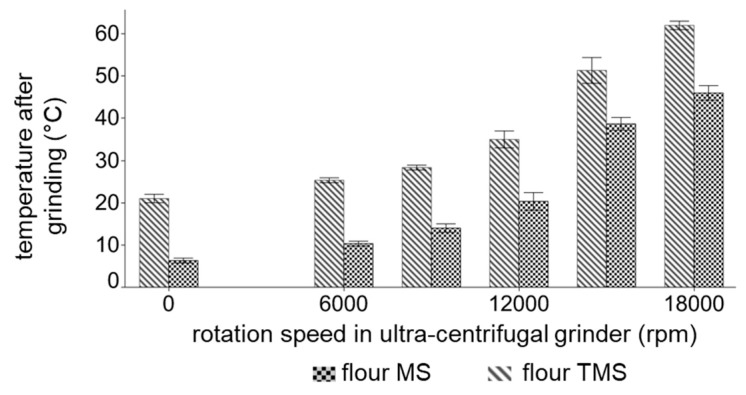
Mean flour temperature after the exposure of mechanical stress in a temperature-controlled impact grinder (flour MS) and after exposure of thermo-mechanical stress in a non-temperature-controlled ultra-centrifugal grinder (flour TMS. Error bars represent standard deviations; *n* = 3.

To elucidate the impact of thermal stress in combination with mechanical stress on flour particle size, wheat flour was either subsequent ground in a temperature-controlled (isolated mechanical stress, MS)) or non-temperature-controlled (mechanical-thermal stress, TMS) impact grinder. The temperature development during MS and TMS processes is illustrated in Figure 3. During non-temperature-controlled TMS treatment, temperatures were raised above 60 °C, when high-rotation speeds were set. The usage of a precooled grinder and flours, however, impeded a global temperature increase above protein denaturation temperatures, which are stated at ~55 °C [28], although local peak temperatures above 60 °C could not be excluded during grinding.

No differences between particle size distribution of exclusively mechanical stress (MS) and thermomechanical stress (TMS) were noticed (compare Figure 2B). Thus, the particle size reduction is a process driven by mechanical forces during grinding and it can be concluded that temperature development has no impact on particle size reduction during grinding.

### 3.2. Starch Modification Degree

**Figure 4:**
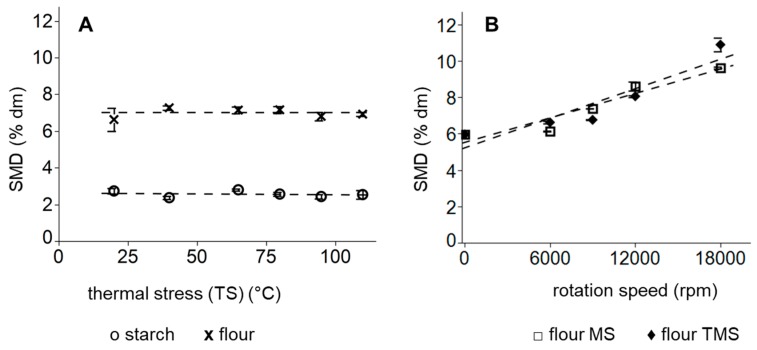
Mean starch modification degree after (A) exclusively thermal treatment of flour x and starch O and (B) after mechanical treatment with thermal stress (TMS) ♦ or without thermal stress (MS) ≤ of wheat flours. Error bars represent standard deviations; *n* = 3.

Beside the particle size distribution of flours, the starch modification degree (SMD) is an important factor for the evaluation of flour quality, as is the baking capability. The measurement of the starch modification degree (SMD) is based on the enzymatic digestibility of starch in flour particles by amylolytic enzymes. Thus, the accessibility and surface/volume ratio of particles significantly affect the SMD. The fragmentation of particles leads to the enlargement of the particle surface, which in turn promotes the digestion by enzymes [5,29,30]. This negative correlation between the particle size distribution and the starch modification degree (SMD) was also confirmed in the present study: while an exclusively thermal treatment of starch and flour did not result in an enhanced starch modification degree (SMD) and or the shift of PSD to smaller sizes (Figure 4A), mechanical treatments with/without thermal forces (MS and TMS) led to a decrease in particle size of wheat flours and an increase in SMD (compare Figure 4B), which is in agreement with other studies, analyzing the effects of grinding on starch modification and particle size [31,32].

Isolated mechanical stress (MS) in temperature-controlled grinder did not lead to differences in SMD in comparison to thermo-mechanical stress (TMS) in non-temperature-controlled grinder at low rotation speed, where a low heat development took place. However, grinding in the ultra-centrifugal grinder at higher rotation speeds (18,000 rpm) resulted in a significantly lower SMD of mechanically stressed flours in comparison to thermo-mechanically stressed flours. Since the combination of thermal and mechanical forces during grinding caused a significantly higher starch modification degree (SMD) as isolated mechanical forces, TMS flours had a higher accessibility of starch for an enzymatic digestion, resulting in altered functional properties during fermentation.

Reasons for the enhanced enzymatic degradation (higher SMD) could be due to transformations in amorphous and the crystalline structures [33,34] caused by intense heat development during non-temperature-controlled grinding. DSC measurements were performed to determine changes in crystalline regions caused by thermal stress.

### 3.3. Gelatinization Properties

The recorded results of the transition of starch crystallites into an amorphous structure (gelatinization properties) of thermally stressed (TS) pure starch and wheat flour are summarized in Table 1. Both the gelatinization enthalpy (Δ*H*)—measure for the degree of starch crystallinity or rather the transition of crystal structure into amorphous structure—and the gelatinization onset—starting temperature of transition of crystalline into amorphous structure—are important parameters in predicting the functional properties of flours. In this present investigation, no continuous changes in gelatinization enthalpy of thermally treated flour or isolated starch were noticed with rise in temperature of TS (Table S1), probably since the gelatinization process of starch requires a minimum water level (for corn starch ≥ 21%) at current process conditions, so that crystalline parts can be transformed into amorphous structures, which depends on treatment conditions prior to the analysis [35]. Unexpectedly, already pure, reference starch showed a lower gelatinization enthalpy (3.02 ± 0.01 J/g dm) than flour (5.63 ± 0.66 J/g dm). Reduced gelatinization enthalpy of isolated starch could be evoked by an exposure of starch to intensive heat and mechanical stress during the commercial extraction procedure, which causes the destruction of crystalline regions of starch. Furthermore, during starch extraction, A granules are predominantly extracted [36] showing a lower relative crystallinity than B granules [37].

However, a steady increase in the gelatinization onset was determined for thermally treated wheat flours (Table 1). Alterations of gelatinization temperature without changes of gelatinization enthalpy are also known from the common process HMT (heat moisture treatment). HMT takes place under low moisture conditions (≤ 20%), leading to an increase in the thermal stability of cereal starches, too [38]. The extent of evoked alterations in gelatinization of starch depends on the heat treatment conditions, as well as the time and constitution of the material. The amylose/amylopectin ratio, the phosphorylation degree, as well as the moisture content of the suspension play an important role in the gelatinization process [35,39,40,41]. In contrast to the present findings, the formation of more stable crystalline structures after HMT [38] alters the gelatinization peak temperature *T*_peak_ of starches, however, *T*_onset_ seems to be less affected by heat-moisture treatments [38]. In particular, the low heating period in this present investigation in comparison to other studies [42,43] makes an increased gelatinization onset due to a restructuration of starch unlikely.

**Figure 5:**
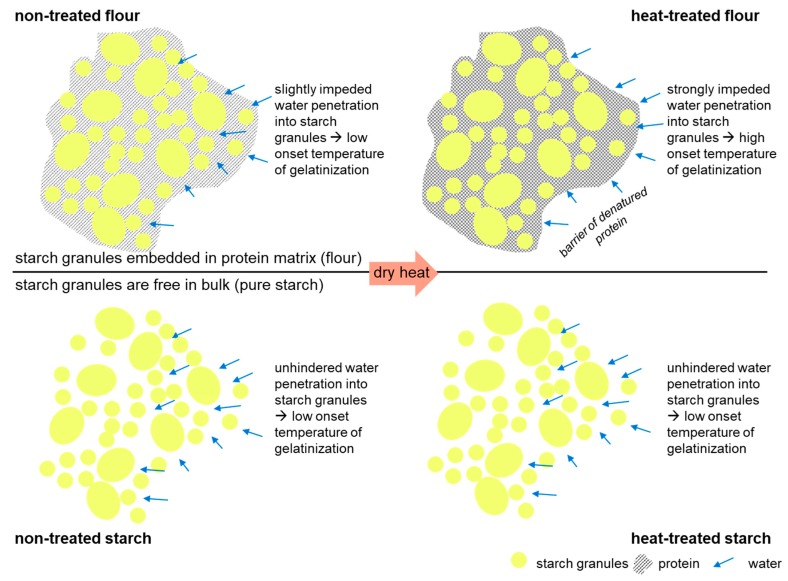
Schematic illustration of the formation of a protein barrier in heat treated wheat matrices and resulting water shielding effect.

Furthermore, a rise in gelatinization onset was solely noticed for wheat flour, although the gelatinization onset of pure starch remained constant. Thus, a reorganization of crystalline parts of starch by heat treatment is unlikely. Rather, it is assumed that changes in gelatinization onset are due to alterations of water distribution between starch and gluten caused by a water barrier of denatured protein, as illustrated schematically in Figure 5. Functional behavior of starch in the matrix ‘flour’ differs from isolated starch. In flour, starch granules are partly isolated from water due to the formation of starch-gluten particles with a higher particle size diameter. The surface of starch granules is covered with small proteins, which significantly affect the overall properties of starch [44]. Those proteins (and lipids), as well as gluten proteins are known to decelerate digestion processes by acting as a natural barrier [45]. Heat treatment of proteins resulted in a formation of agglomerates, possibly due to a formation of disulphide bonds [27]. It is postulated that these newly formed bonds do strengthen protein cohesion, resulting in a merging of the protein cover layer and a more impeded water penetration. Consequently, water penetration is retarded, and higher temperatures are necessary to induce a water penetration into starch-gluten agglomerates. Nuclear magnetic resonance measurements should therefore be performed to finally show the water barrier effects of heat-treated proteins.

On the other hand, starch granules of pure starch are freely accessible for water, since proteins were removed during the extraction of pure wheat starch. Consequently, heat treatment does not affect the gelatinization onset of starch. To investigate in more detail changes in polymer functionality due to the different physical treatments, water retention capacity (WRC) and dough functionality were analyzed.

### 3.4. Functional Alterations of Cereal Polymers

**Figure 6:**
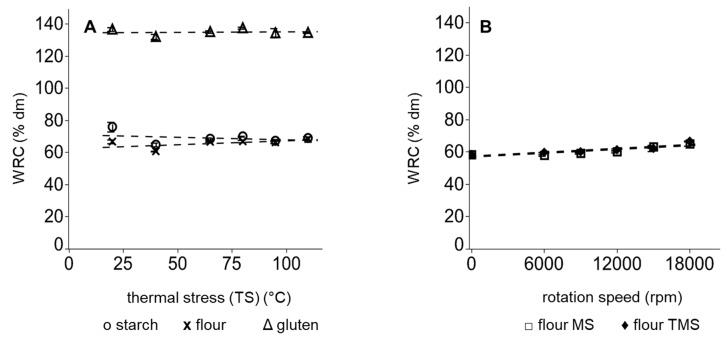
Mean water retention capacity after (**A**) exclusively thermal treatment of flour x, starch O and gluten Δ and (**B**) after mechanical treatment with thermal stress (TMS) ♦ or without thermal stress (MS) ≤ of wheat flours. Error bars represent standard deviations; *n* = 3

In the baking industry, the hydration properties of wheat flour are of special interest in determining the dough processability and affecting the quality of bread [46]. As shown in Figure 6A, isolated thermal stress (TS) did not modify significantly the water retention capacity (WRC) of heat-treated gluten, starch or flour. WRC of MS and TMS wheat flours was raised with an increase in the rotation speed of an ultra-centrifugal grinder (up to 18,000 rpm) from 58.61 ± 0.82% dm to 65.94 ± 1.82% dm and 66.62 ± 0.38% dm, respectively. Elevated hydration properties of flours after grinding are explainable by the reduction in particle size and consequently the enhancement of the surface of flour particles. Furthermore, alterations in chemical bonds of the surface of flour particles can occur, leading to a rise in hydrophilic bonds [5,47]. Since MS and TMS showed same effects on WRC, alterations in hydration properties of starch in excess water are induced solely by a mechanical modification of flours.

These insights bring advantages for the construction of modern mills and the control of grinding processes, since flour functionalities and qualities can be set during grinding, depending on the purposed usage. If hydration properties are in the foreground, heat development during grinding can be neglected and consequently costs for cooling units can be economized. However, if the gelatinization properties are relevant, for instance in flours for bread production, heat development in grinders should be controlled during the grinding procedure to achieve a standardization of the flour quality.

However, results from the applied WRC method (AACC 56–11.02), which determine the hydration properties of flours in excess water, cannot be directly transferred to a matrix with limited water content, as for doughs [48]. To investigate hydration properties under limited water content and to elucidate alterations in dough characteristic of physically modified flours, a Z-kneading system was additionally used. The results are summarized in Table 2.

Water absorption of thermally stressed flours increased slightly from 61.0 ± 0.3% (+20 °C) to 62.0 ± 0.3 (+110 °C) suggesting a modification of wheat polymers. Significant alterations occurred solely for a temperature of 110 °C, hence above the denaturation temperature of proteins. This denaturation can result in a higher water binding capacity of proteins and consequently firmer dough, if the water amount is not adapted. Since WRC is performed in excess water, slight changes in hydration properties might not be visible. However, even small alterations in hydration properties may significantly alter the functional properties of doughs. The dough development times for TS flours stayed constant for temperature up to 110 °C.

To summarize, in limited water systems (DoughLab) and as well in excess water (DSC gelatinization onset), the impact of thermal treatment on polymer functionality was visible. The onset temperature and furthermore the water absorption was elevated. Thus, thermal stresses of flours during grinding or transportation of flours influence the processing of wheat dough and the manufacturing of wheat products. Consequently, temperatures during the processing of grains, the transportation and storage of flours should be controlled to a enable a constant flour quality.

However, wheat functionality and the ability to enclose high amounts of gas is mainly attributed to the unique properties of the gluten network. To clarify if findings are generally valid for further cereals, TS (thermal stress), MS (mechanical stress) and TMS (thermomechanical stress) was applied to rice, rye and a second wheat variety (Table 3).

### 3.5. Transferability to Other Cereal Flours

In accordance to previous findings, TS did not affect WRC or SMD of a second wheat flour (wheat 2). Thus, results from previous chapters are transferable to other wheat varieties.

However, thermal stress of rice and rye flour caused a rise in WRC from 95.97 ± 0.68% dm to 104.58 ± 0.70% dm and from 124.74 ± 0.30% dm to 135.60 ± 0.97% dm, respectively. Interestingly, TS of rice and rye flour caused higher WRC, but did not elevate the starch modification degree. Since accessibility of starch is not altered by TS, a higher amount of water is retained by non-starch components of rice and rye flours. Further research should focus in detail on the alterations of proteins and arabinoxylans from rice and rye caused by thermal stress.

Mechanical treatments (18,000 rpm) with or without thermal stress caused significant alterations of the WRC and SMD within all flours. For rye flours, the combination of thermal and mechanical stress (TMS) caused significantly higher WRC and SMD than pure mechanical stress MS (compare TMS rye and MS rye). The rise of WRC of TMS rye could be explained by the enhanced swelling of heat treated arabinoxylans [49], however, to prove this hypothesis, pure arabinoxylans should be exposed to a dry heat treatment. Thus, flours with additional and influential polymers, as arabinoxylans, should be further analyzed in particular to understand the polymer interactions in detail. In summary, starch modification can be achieved by mechanical treatments in all analyzed cereals, however, in cereals with further functional biopolymers than starch and gluten, modification of all biopolymers should be considered.

## 4. Conclusions

Physical treatments (for instance during grinding) can be used to gain more detailed knowledge of structure-function relations in complex cereal matrices, as wheat flour, and to produce flours with altered functional properties for the use in clean label products. This study showed that functional alteration of wheat starch is exclusively based on mechanical stress during grinding, however, thermal stress can cause a modification of the gelatinization onset of wheat flour. Thus, proteins and/or other components of flours have to exhibit changes in structure or conformation leading to a rise in gelatinization onset and flour hydration. This indicates that wheat proteins are thermally modified even in dry matrices, which necessitates a detailed investigation of the functional alteration of gluten caused by thermo-mechanical processes. Depending on the field of application, thermal forces should therefore be monitored and, moreover, controlled during the grinding process and any further processing of grains.

## Figures and Tables

**Table 1:** Mean (± SD) gelatinization onset *T*onset of thermally treated (TS) wheat flour and wheat starch. SD = standard deviation; *n* = 3.

Thermal Treatment (°C)	*T*_onset_ Flour (°C)	*T*_onset_ Starch (°C)
**+20**	46.62 ± 1.70 ^C^	52.73 ± 0.27 ^A^
**+40**	46.92 ± 0.57 ^B,C^	52.52 ± 0.18 ^A^
**+65**	50.67 ± 1.98 ^A,B,C^	52.24 ± 0.29 ^A^
**+80**	51.97 ± 0.11 ^A,B^	52.35 ± 0.13 ^A^
**+95**	52.81 ± 0.46 ^A^	52.31 ± 0.20 ^A^
**+110**	52.01 ± 0.58 ^A^	52.44 ± 0.14 ^A^

Different letters demonstrate significant differences in columns.

**Table 2:** Mean (± SD) dough water absorption and dough development time of thermally treated wheat flour determined by a Z-kneading system. SD = standard deviation; *n* = 3.

Thermal Treatment (°C)	Water Absorption (%)	Dough Development Time (min)
**+20**	61.0 ± 0.3 ^B^	3.4 ± 0.8 ^A^
**+40**	61.6 ± 0.2 ^A,B^	3.4 ± 0.4 ^A^
**+65**	61.2 ± 0.0 ^A,B^	3.4 ± 0.2 ^A^
**+80**	61.3 ± 0.5 ^A,B^	3.7 ± 0.4 ^A^
**+95**	61.4 ± 0.3 ^A,B^	3.8 ± 1.3 ^A^
**+110**	62.0 ± 0.3 ^A^	4.0 ± 0.4 ^A^

Different letters demonstrate significant differences in columns.

**Table 3:** Impact of thermal stress (TS) at 110 °C, thermal-mechanical stress (TMS) in non-temperature-controlled grinder (18,000 rpm) and mechanical stress (MS) in temperature-controlled grinder (18,000 rpm) on mean (± SD) water retention capacity (WRC) and starch modification degree (SMD) of rice, rye and an additional wheat flour. SD = standard deviation; *n* = 3.

Sample	WRC (% dm)	SMD (% dm)
**Ref rice**	95.97 ± 0.68 ^D^	8.71 ± 0.25 ^C^
**TS rice**	104.58 ± 0.70 ^C^	8.44 ± 0.12 ^C^
**TMS rice**	110.56 ± 1.18 ^B^	14.60 ± 0.12 ^B^
**MS rice**	114.57 ± 1.41 ^A^	15.96 ± 0.28 ^A^
**Ref rye**	124.74 ± 0.30 ^D^	4.67 ± 0.09 ^C^
**TS rye**	135.60 ± 0.97 ^B^	4.64 ± 0.15 ^C^
**TMS rye**	138.85 ± 0.97 ^A^	6.06 ± 0.13 ^A^
**MS rye**	132.95 ± 0.76 ^C^	5.73 ± 0.13 ^B^
**Ref wheat 2**	49.02 ± 0.30 ^B^	6.91 ± 0.21 ^C^
**TS wheat 2**	51.55 ± 3.41 ^A,B^	6.67 ± 0.28 ^C^
**TMS wheat 2**	54.28 ± 1.55 ^A^	7.79 ± 0.39 ^B^
**MS wheat 2**	55.26 ± 0.47 ^A^	8.81 ± 0.14 ^A^

Different letters demonstrate significant differences in columns.

## Supplementary Materials

The following are available online at https://www.mdpi.com/2073-4360/11/3/448/s1. Table S1. Mean (± SD) gelatinization enthalpy ΔH of thermally treated wheat flour and wheat starch. SD = standard deviation; *n* = 3.
