# Supplementary material for: Mechanically and Thermally Induced Degradation and Modification of Cereal Biopolymers during Grinding
Source: Polymers (Basel). 2019 Mar 8;11(3):448. doi: 10.3390/polym11030448 (PMC6473319; doi:10.3390/polym11030448)
Supplement: Supplementary file 1 [file polymers-11-00448-s001.pdf]

Supplementary Materials

Mechanically and Thermally Induced Degradation and Modification of Cereal Biopolymers during Grinding

Sabina Paulik, Mario Jekle* and Thomas Becker

Technical University of Munich, Institute of Brewing and Beverage Technology, Research Group Cereal Technology and Process Engineering, 85354 Freising, Germany; sabina.jakobi@tum.de (S.P.); mjekle@tum.de (M.J.); tb@tum.de (T.B.)

* Correspondence: mjekle@tum.de (M.J.); Tel.: +49-8161-71-3669

Table S1. Mean (± SD) gelatinization enthalpy Δ*H* of thermally treated wheat flour and wheat starch. SD = standard deviation; *n* = 3.

Thermal treatment (°C)	Δ*H* flour (J/g dm)	Δ*H* starch (J/g dm)
**+20**	5.63 ± 0.66 [A,B]	3.02 ± 0.01 [A]
**+40**	6.22 ± 0.31 [A]	2.95 ± 0.12 [A]
**+65**	5.60 ± 0.41 [A,B]	2.91 ± 0.09 [A]
**+80**	5.30 ± 0.37 [A,B]	2.97 ± 0.49 [A]
**+95**	4.28 ± 0.17 [B]	2.69 ± 0.03 [A]
**+110**	5.68 ± 0.39 [A]	2.83 ± 0.11 [A]
